# Prevalence of anxiety in parents of Children with Adrenal insufficiency: a case control study

**DOI:** 10.1192/j.eurpsy.2022.983

**Published:** 2022-09-01

**Authors:** N. Faouel, R. Ben Soussia, K. Messai, M. Kacem, W. Bouali, A. Haj Mohamed, L. Zarrouk

**Affiliations:** 1 hospital Tahar sfar Mahdia, Epartment Of Psychiatry Mahdia, Mahdia, Tunisia; 2 hospital Tahar sfar Mahdia, Department Of Psychiatry Mahdia, Mahdia, Tunisia; 3 Hospital Tahar sfar Mahdia, Department Of Psychiatry Mahdia, Mahdia, Tunisia; 4 University Hospital of Mahdia, Tunisia., Psychiatry, mahdia, Tunisia; 5 hospital Tahar sfar Mahdia, Department Of Psychiatry Mahdia, monastir, Tunisia

**Keywords:** Anxiety, adrenal insufficiency, Children, parents

## Abstract

**Introduction:**

Adrenal insufficiency is a rare medical condition which can occur in children. Parents, being the primary support and support for the child, are generally involved in the care of their sick child.

**Objectives:**

To estimate the prevalence of anxiety symptoms and associated factors in parents of children with adrenal insufficiency.

**Methods:**

This is an analytical cross-sectional case-control study over a period of 4 months in 2019, carried out with parents of children with Adrenal Insufficiency followed at the pediatric outpatient clinic in Taher Sfar Mahdia University Hospital. We used an anonymous questionnaire that included a socio-demographic fact sheet and the Hamilton anxiety scale for exploring anxiety symptoms.

**Results:**

A total of 38 parents of children with Adrenal insufficiency and 38 control parents participated in the study. The current age of the child was between 1 and 16 years old with an average of 9.1 and standard deviation of 4.22 For the assessment of anxiety, 55.3% of the parents had a score greater than 20 attesting to the presence of an anxiety symptomatology. In addition, only 26.3% of control parents presented anxious symptoms. There is a significant difference between the two populations (p = 0.010 OR = 3.459). Anxiety was associated with having a child with SI (OR=3.4), female gender (OR=4.2), unemployment (OR=6.33), and low socioeconomic status.

**Conclusions:**

Parents have a considerable burden in the care and management of their child with a chronic illness, which takes time and a lot of patience. Detecting anxiety symptoms in this population will help them manage it.

**Disclosure:**

No significant relationships.

